# Methodological problems with the test of the Paleo diet by Lamont *et al.* (2016)

**DOI:** 10.1038/nutd.2016.22

**Published:** 2016-06-27

**Authors:** N Cofnas

**Affiliations:** 1 Department of History and Philosophy of Science, University of Cambridge, Free School Lane, Cambridge, CB2 3RH, UK

Some studies of the twentieth-century hunter–gatherer diets have found them to be low in carbohydrates and high in fat, relative to the Department of Agriculture's dietary guidelines.^1, 2, 3^ Although there is a disagreement regarding the carbohydrate content of the ancestral human diet,^4^ advocates of Paleolithic diets who hold that high-carbohydrate foods were not frequently eaten by our pre-agricultural ancestors have recommended low-carbohydrate high-fat diets (LCHFDs) as a way to possibly prevent obesity and ameliorate some diseases, including diabetes.^1, 2^

Lamont *et al.*^5^ recently attempted to test this version (that is, the LCHF version) of the Paleo diet. They fed prediabetic New Zealand obese mice either LCHFDs (carbohydrates comprising 6% of energy, 81% of fat) or standard rodent chow (carbohydrates comprising 70% of energy, 10% of fat). Mice in the former (that is, the experimental) condition experienced ‘greater weight gain and insulin resistance.' Although Lamont *et al.* do not mention the Paleo diet by name in the paper, in an interview with ABC News the lead author (Andrikopoulos) claims that the study suggests that ‘The Paleo diet may not necessarily be good for everybody.' He was paraphrased as saying that ‘the results of [the] study were a cautionary tale about fad diets.'^6^ A YouTube video released by the authors' university is titled simply ‘Paleo diets=weight gain.'^7^ But Lamont *et al.*'s results do not undermine the theory behind the Paleo diet. Their results are exactly what would be predicted by the theory. Why is that?

The theory behind the Paleo diet is that each species is genetically adapted to thrive on the diet eaten by its ancestors throughout most of the species' evolutionary history.^8^ Consequently (according to the theory) members of a species will have the best health outcomes when they eat a diet that resembles the ancestral one in terms of its basic properties (the ratio of macronutrients, amounts of various vitamins and so on). They will have poorer health outcomes if they eat a diet with radically different properties—on the view that the human ancestral diet was LCHF, *Homo sapiens* will have poorer health outcomes on diets high in carbohydrates and low in fat.

Paleo theory predicts that mice will flourish most on their ancestral diet. Mice (*Mus musculus*) are omnivores, but studies of the stomach contents of mice in the wild suggest that, though their diet varies by region and time of year, it is composed largely of seeds and other plant material high in carbohydrates, and does not contain any high-fat food sources.^9^ The rodent chow fed to the mice in Lamont *et al.*'s control condition was high in carbohydrates and low in fat—in this respect it was similar to the mouse ancestral diet. Therefore, it was the mice in the control condition that were actually fed something close to a (mouse) Paleo diet. Mice in the experimental condition were fed something loosely based on a version of the human Paleo diet, which for mice is not Paleo. Although the results of Lamont *et al.* do not say anything about health outcomes for humans on a (human) Paleo diet, their results are consistent with Paleo theory in that they confirm one of its predictions: they demonstrate that mice put on a diet deviating significantly from their ancestral one experienced worse health outcomes. Studies on humans, however, suggest that for that species LCHFDs are beneficial in diabetes management.^10^
